# The Clinical Utility of Two High-Throughput 16S rRNA Gene Sequencing Workflows for Taxonomic Assignment of Unidentifiable Bacterial Pathogens in Matrix-Assisted Laser Desorption Ionization–Time of Flight Mass Spectrometry

**DOI:** 10.1128/JCM.01769-21

**Published:** 2022-01-19

**Authors:** Hiu-Yin Lao, Timothy Ting-Leung Ng, Ryan Yik-Lam Wong, Celia Sze-Ting Wong, Lam-Kwong Lee, Denise Sze-Hang Wong, Chloe Toi-Mei Chan, Stephanie Hoi-Ching Jim, Jake Siu-Lun Leung, Hazel Wing-Hei Lo, Ivan Tak-Fai Wong, Miranda Chong-Yee Yau, Jimmy Yiu-Wing Lam, Alan Ka-Lun Wu, Gilman Kit-Hang Siu

**Affiliations:** a Department of Health Technology and Informatics, The Hong Kong Polytechnic University, Hong Kong Special Administrative Region, China; b Department of Clinical Pathology, Pamela Youde Nethersole Eastern Hospital, Hong Kong Special Administrative Region, China; Mayo Clinic

**Keywords:** 16S rRNA gene, bacterial species, Illumina sequencing, Nanopore sequencing, Sanger sequencing

## Abstract

Bacterial pathogens that cannot be identified using matrix-assisted laser desorption ionization–time of flight mass spectrometry (MALDI-TOF MS) are occasionally encountered in clinical laboratories. The 16S rRNA gene is often used for sequence-based analysis to identify these bacterial species. Nevertheless, traditional Sanger sequencing is laborious, time-consuming, and low throughput. Here, we compared two commercially available 16S rRNA gene sequencing tests that are based on Illumina and Nanopore sequencing technologies, respectively, in their ability to identify the species of 172 clinical isolates that failed to be identified by MALDI-TOF MS. Sequencing data were analyzed by the respective built-in programs (MiSeq Reporter software of Illumina and Epi2me of Nanopore) and BLAST+ (v2.11.0). Their agreement with Sanger sequencing on species-level identification was determined. Discrepancies were resolved by whole-genome sequencing. The diagnostic accuracy of each workflow was determined using the composite sequencing result as the reference standard. Despite the high base-calling accuracy of Illumina sequencing, we demonstrated that the Nanopore workflow had a higher taxonomic resolution at the species level. Using built-in analysis algorithms, the concordance of Sanger 16S with the Illumina and Nanopore workflows was 33.14% and 87.79%, respectively. The agreement was 65.70% and 83.14%, respectively, when BLAST+ was used for analysis. Compared with the reference standard, the diagnostic accuracy of Nanopore 16S was 96.36%, which was identical to that of Sanger 16S and better than that of Illumina 16S (69.07%). The turnaround time of the Illumina workflow and the Nanopore workflow was 78 h and 8.25 h, respectively. The per-sample cost of the Illumina and Nanopore workflows was US$28.5 and US$17.7, respectively.

## INTRODUCTION

Traditionally, clinical microbiology laboratories have relied on phenotypic methods to identify bacterial pathogens. However, conventional biochemical tests are labor-intensive and time-consuming, and the results can be ambiguous when two species share similar biochemical profiles (1, 2). Nowadays, matrix-assisted laser desorption–ionization time of flight mass spectrometry (MALDI-TOF MS) is widely used for bacterial identification in clinical laboratories (3). MALDI-TOF MS allows rapid identification of microorganisms by comparing the mass spectrum of a sample with the reference spectra in the database (4). Although MALDI-TOF MS is a rapid, simple, and high-throughput technology for bacterial identification, some species cannot be well differentiated due to high similarity in the mass spectra of closely related species or lack of reference spectra (5).

A study by Lau et al. reported that MALDI-TOF MS failed to determine the species of 37 out of 67 (55%) phenotypically “difficult-to-identify” bacteria in clinical laboratories (6). In general, anaerobes, particularly *Actinomyces* spp., *Peptostreptococcus* spp., *Prevotella* spp., and *Fusobacterium* spp. (7–9), have a higher failure rate than aerobes in bacterial identification using MALDI-TOF MS (7, 10). Additionally, some weakly acid-fast bacilli and Gram-positive aerobes, such as *Nocardia* spp. and *Streptomyces* spp., respectively, are poorly identified by MALDI-TOF MS (7, 11). Regarding Gram-negative aerobes, studies have reported that MALDI-TOF MS cannot effectively identify certain *Achromobacter* spp., Acinetobacter spp., *Chryseobacterium* spp., and *Moraxella* spp. (11, 12). In such cases, 16S sequencing of cultured isolates is commonly used for species-level identification.

Sanger sequencing offers a high base-calling accuracy, but it is laborious and time-consuming with limited throughput (13). High-throughput sequencing (HTS) technologies, such as Illumina sequencing and Nanopore sequencing, have been proposed as alternatives to generate 16S sequences for rapid identification of bacteria that are of clinical interest. The Illumina platform can generate vast quantities of highly accurate sequencing reads. However, the read length is limited and insufficient to cover the entire 16S rRNA gene. According to the 16S metagenomic sequencing library preparation workflow from Illumina, bacteria are identified based on variable regions (V3 and V4) of 16S. Nevertheless, the variable regions are not equally discriminative between and across different species, genera, and families (14).

In contrast, the MinION device by Oxford Nanopore Technologies (ONT) enables generation of ultralong reads exceeding 4 Mb. The 16S rRNA sequencing assay (SQK-16S024) from ONT allows the entire 16S rRNA gene to be sequenced with real-time data analysis. Recent studies have demonstrated its potential for rapid bacterial identification; however, the high read error rate (8% to 15%) of this platform might hinder the accuracy of species-level identification for diagnostic purposes (15).

Considering the respective limitations of Illumina and Nanopore technologies, a comprehensive investigation of the clinical utility of these 16S rRNA sequencing approaches for bacterial identification is required. This study aimed to evaluate the performance of two commercial HTS workflows for 16S rRNA sequencing, which were the 16S metagenomic sequencing library preparation workflow (Nextera XT index kit v2) coupled with MiSeq Reporter software (MSR) from Illumina and the 16S barcoding kit 1-24 (SQK-16S024) coupled with Epi2me from ONT. These workflows were used to identify bacterial isolates that could not be definitively identified by MALDI-TOF MS. The respective performances of the two built-in analysis pipelines (MSR and Epi2me) were compared with that of the in-house BLAST+ (v2.11.0) analysis.

In light of the complexities of evaluating diagnostic accuracy in the absence of a perfect gold standard, we considered a composite 16S rRNA sequencing result inferred by Sanger and the two HTS platforms as a reference standard. In cases of disagreement in taxa inferred by the three sequencing platforms, whole-genome sequencing (WGS) was conducted to confirm the bacterial identities. The costs and times to result of the sequencing workflows were also compared.

## MATERIALS AND METHODS

### Sample collection.

A total of 172 clinical isolates from 117 species were collected from the clinical microbiology laboratory of Pamela Youde Nethersole Eastern Hospital in Hong Kong. Clinical isolates were included if they failed to be classified at the species level (score < 2.00) by the IVD MALDI Biotyper (Bruker Daltonics, Bremen, Germany). The MALDI-TOF MS procedures were repeated twice to eliminate the effect of random errors. Failure to identify bacterial species occurred due to (i) lack of a reference spectrum in the database (81 samples), (ii) inclusion of certain species in the “dangerous database,” named Security Library 1.0, rather than the regular database (two samples), or (iii) poor quality of protein spectra (89 samples) (see Table S1 in the supplemental material). The IVD MALDI Biotyper used in this study was microflex (Bruker Daltonics), and the database version was BD-6763. The original specimens from which the organisms were isolated are listed in Table S1 in the supplemental material.

### DNA extraction.

Total nucleic acid was extracted from clinical isolates using the Amplicor respiratory specimen preparation kit (Roche, Basel, Switzerland) and purified with 1.8× AMPure XP beads (Beckman Coulter, CA, USA). Purified DNA was diluted to targeted concentrations in subsequent sequencing workflows. The required DNA inputs for the Illumina and Nanopore workflows were 12.5 ng and 10 ng, respectively.

### Sanger 16S.

For Sanger 16S rRNA sequencing (Sanger 16S), the full-length 16S rRNA gene was amplified using primers for 16s_27F (5′-AGAGTTTGATCMTGGC-3′´) and 16s_1492R (5′-TACCTTGTTACGACTT-3′´) (Fig. S1) (16). The reaction mixture was prepared by mixing 36.7 μL of nuclease-free water, 5 μL of 10× PCR buffer, 1 μL of 10 mM deoxynucleoside triphosphate mix (NEB, Ipswich, MA, USA), 1 μL of each 25 μM primer, 0.3 μL of HotStarTaq Plus DNA polymerase (Qiagen, Hilden, Germany), and 5 μL of DNA template. The PCR conditions were 96°C for 8 min, 37 cycles at 94°C for 1 min, 37°C for 2 min, and 72°C for 2 min 30 s, followed by 72°C for 10 min and a hold step at 4°C. PCR products were purified using ExoSAP-IT reagent (Thermo Fisher Scientific, Waltham, MA, USA) and then passed to the subsequent cycle sequencing by using eight sequencing primers (17–19) (Table S2). The reaction mixture consisted of 13 μL of nuclease-free water, 1 μL of BigDye Terminator v3.1 ready reaction mix (Thermo Fisher Scientific), 3.5 μL of 5× sequencing buffer, 1 μL of 3.2 μM primer, and 1.5 μL of purified PCR product. The PCR conditions were 96°C for 1 min and 25 cycles at 96°C for 10 s, 37°C for 30 s, and 60°C for 4 min, followed by a hold step at 4°C. The sequencing products were purified using 75% isopropanol and resuspended in 12 μL of Hi-Di formamide (Thermo Fisher Scientific). After loading on the Applied Biosystems 3130 genetic analyzer (Thermo Fisher Scientific), the resulting raw trace files were analyzed using the Staden Package (v2.0.0b11). The consensus sequence of each sample was classified by submitting a Basic Local Alignment Search Tool (BLAST) query against the 16S rRNA sequence database (https://blast.ncbi.nlm.nih.gov/Blast.cgi), using the default parameters. The classified species with the lowest E value and highest percentage identity was regarded as the identity of the sample.

### Illumina 16S. (i) Library preparation.

For Illumina sequencing (Illumina 16S), libraries were constructed according to the 16S metagenomic sequencing library preparation workflow from Illumina. Briefly, the 16S V3 and V4 regions of samples were amplified in the first stage of PCR using the primers suggested in the workflow, which were 16S amplicon PCR forward primer (5′-TCGTCGGCAGCGTCAGATGTGTATAAGAGACAGCCTACGGGNGGCWGCAG-3′) and 16S amplicon PCR reverse primer (5′-GTCTCGTGGGCTCGGAGATGTGTATAAGAGACAGGACTACHVGGGTATCTAATCC-3′) (Fig. S1). The underlined bases in the primer sequences are the overhang adapter sequences for attachment of the indexed adapters in the second stage of PCR. The size of the amplicon was approximately 460 bp. After a post-PCR cleanup, a unique indexed sequencing adapter was added to each sample using the Nextera XT index kit v2 (Illumina, San Diego, CA, USA). Then, a second post-PCR cleanup was performed, followed by a qualification check of the purified libraries.

### (ii) Quantification and sequencing.

The size of each library was measured using the 2100 Bioanalyzer system (Agilent, Santa Clara, CA, USA) and the high-sensitivity DNA kit (Agilent). The quantity of the libraries was measured by real-time PCR using the LightCycler 480 instrument II (Roche) and the QIAseq Library Quant assay kit (Qiagen). Then, the libraries were diluted to 4 nM and pooled into one tube. After denaturation with 0.2 N NaOH, the pooled library was diluted to 9 pM and spiked with 15% of 9 pM PhiX prepared from the PhiX control kit v3 (Illumina). The pooled library was then loaded on the MiSeq sequencer (Illumina) for sequencing using MiSeq reagent kit v3 (Illumina). The sequencing time was 56 h.

### (iii) On-instrument data analysis.

Sequencing data were analyzed using MiSeq Reporter software (v2.6.2.3) (MSR) in the MiSeq system. After selection of the metagenomics workflow, sequencing reads were mapped against reference sequences in the Greengenes database (v13.5, May 2013) (http://greengenes.lbl.gov/) for classification. The classification of reads at seven taxonomic levels from kingdom to species was analyzed in this workflow.

### (iv) Data analysis using BLAST+ (Illumina_BLAST+).

The paired-end reads of each sample were merged using the “make.contigs” command in Mothur (v1.44.3) (20). The reads were filtered using the “screen.seqs” command. Sequences smaller than 400 bp, larger than 500 bp, or with any ambiguous bases were removed. The resulting fasta files were analyzed by BLAST+ (v2.11.0) using an in-house Python script (https://github.com/siupenyau/Pocket_16S/tree/7d3fa9d73a6a35afb47e40e7850cef72b4b91a22). In brief, the reads were aligned to the reference sequences in the 16S rRNA database (https://ftp.ncbi.nlm.nih.gov/blast/db/) downloaded from the National Center for Biotechnology Information (NCBI). The percentage identity and percentage query coverage were set at 90%.

### (v) Data analysis using nf-core/ampliseq.

Samples with disagreements between the MSR and Illumina_BLAST+ were further analyzed using another pipeline, nf-core/ampliseq (https://github.com/nf-core/ampliseq), which was developed by Straub et al. (21) to obtain the resolved Illumina 16S identity. The pipeline performed taxonomic assignments based on an error-correcting amplicon sequence variant (ASV) approach instead of read-by-read classification. The reference 16S rRNA database was the SILVA v132 database (22).

### Nanopore 16S. (i) Library preparation and sequencing.

For Nanopore sequencing (Nanopore 16S), library preparation was performed using the 16S barcoding kit 1-24 (SQK-16S024) from ONT according to the manufacturer’s protocol. Libraries were quantified using the Qubit 2.0 fluorometer (Thermo Fisher Scientific) with the Qubit 1× double-stranded DNA (dsDNA) HS assay kit (Thermo Fisher Scientific). Then, 24 barcoded libraries were pooled into one tube in equal concentrations. After ligation with the rapid adapter, sequencing was performed using the FLO-MIN106 R9.4.1 flow cell with the MinION sequencer on the MinKNOW platform for approximately 4 h.

### (ii) On-instrument real-time data analysis.

During sequencing, the passed fastq files generated by Guppy of MinKNOW, which had a quality score of >7, were uploaded on the cloud-based data analysis platform Epi2me for analysis. Sequencing reads were aligned to reference sequences in the NCBI 16S bacterial database using the FASTQ 16S workflow (v2020. 04. 06). Regarding the workflow parameters, the minimum QSCORE was set at 7, while the minimum percentage coverage and minimum percentage identity were set at 90%.

### (iii) Data analysis using BLAST+ (NanoBLAST+).

In addition to Epi2me, sequencing data were analyzed using BLAST+ (v2.11.0), similar to the analysis of Illumina data. As each sample generated multiple fastq files in a sequencing run, the fastq files of each sample were first merged into a single fastq file and then converted to a fasta file before being aligned to reference sequences in the database.

### (iv) Data analysis using NanoCLUST.

Samples with disagreement between Epi2me and NanoBLAST+ were further analyzed using another pipeline, NanoCLUST (https://github.com/genomicsITER/NanoCLUST) (23), to obtain the resolved Nanopore 16S identity. Unlike Epi2me and NanoBLAST+, NanoCLUST does not classify individual reads in a sample. Instead, NanoCLUST forms clusters of similar reads and classifies the consensus sequence of each cluster.

### WGS.

Samples with completely discordant taxa, as inferred by Sanger 16S, Illumina 16S, and Nanopore 16S tests, were subjected to whole-genome sequencing (WGS) to confirm the definite identities using the ONT platform. Library preparation was performed using the transposase-based rapid barcoding kit (SQK-RBK110.96) from ONT in accordance with the manufacturer’s protocol. After pooling and adapter ligation, the library was loaded on the FLO-MIN106 R9.4.1 flow cell and sequenced using the GridION device for 48 h in high-accuracy base-calling mode. The passed fastq files were uploaded to Epi2me and analyzed using the WIMP workflow (v2021.03.05).

### *De novo* assembly for WGS data sets.

Sequencing reads of each sample were assembled using Shasta (v0.7.0) (https://github.com/chanzuckerberg/shasta). Sequencing reads were realigned to the assembled consensus sequences using minimap2 (v2.17-r941) and samtools (v1.10). Consensus sequences were first polished using MarginPolish (v1.3.dev-5492204) (https://github.com/UCSC-nanopore-cgl/MarginPolish) and then further polished using homopolish (v0.2.1) (https://github.com/ythuang0522/homopolish) (24). To avoid bioinformatics bias in *de novo* assembly, each sample was also subjected to a second analysis pipeline. In brief, the sequencing reads were assembled using miniasm (v0.3-r179) (https://github.com/lh3/miniasm/releases/tag/v0.3). All-versus-all read self-mapping was performed using minimap2. Raw consensus sequences were then generated using miniasm. After realignment of the raw reads to consensus sequences using minimap2, the consensus sequences were polished twice using racon (v1.4.3) (https://github.com/isovic/racon).

The longest polished consensus sequences of each sample were classified using BLAST+ (v2.11.0) with the Prokaryotic RefSeq Genomes database downloaded from the NCBI. The top classified species with both query coverage and percentage identity were reported. The average nucleotide identity (ANI) between the query and best-matched reference genomes was calculated using an ANI calculator (https://www.ezbiocloud.net/tools/ani) (25). An ANI of >94% indicated that the samples belong to the same species as the best-matched genomes.

### Data and statistical analysis.

The top classified taxa obtained from Illumina and Nanopore data sets were compared with those inferred by Sanger 16S using built-in programs and BLAST+ for analysis. Species-level concordance between the HTS and Sanger workflows was calculated. For samples that did not match at the species level, concordance at the genus or family level was determined.

To assess diagnostic accuracy, a composite 16S rRNA sequencing result obtained from the three sequencing platforms was considered the reference standard. Identical species obtained by at least two sequencing platforms were considered reference taxa. For samples with completely discordant species inferred by the three sequencing platforms, WGS was conducted to confirm the reference taxa.

## RESULTS

### Statistics of sequencing reads generated from the Illumina and Nanopore workflows.

Based on the default analysis of MSR, the Illumina platform generated an average of 113,381 reads per sample. After merging the paired-end reads and filtering out unwanted reads with undesired read lengths and ambiguous bases, an average of 68,652 filtered reads per sample was retained for Illumina_BLAST+ analysis.

The Nanopore MinKNOW platform generated an average of 51,769 reads (QSCORE ≥ 7) per sample, but an average of 51,419 reads (QSCORE ≥ 7) per sample was analyzed in the FASTQ 16S workflow in Epi2me. The slight difference in the number of average reads per sample was due to using different algorithms in the demultiplexing step between Epi2me and Guppy of MinKNOW. An average of 51,769 reads per sample was analyzed using NanoBLAST+. The total number of reads and the number of classified reads of each sample on both sequencing platforms are shown in Table S3 in the supplemental material.

### Taxonomic resolution of sequencing reads.

**FIG 1:**
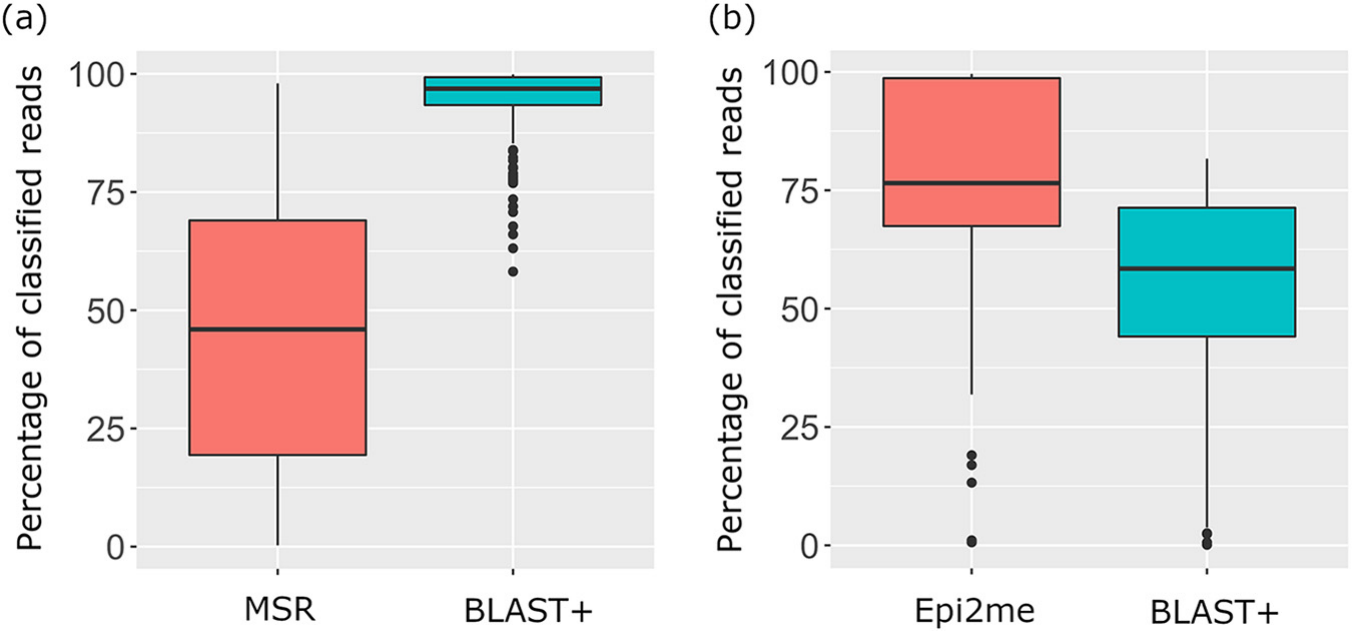
Boxplots showing the distribution of percentages of classified reads of all samples in Illumina (a) and Nanopore (b) sequencing.

The percentage distribution of classified reads via both sequencing platforms is shown in Fig. 1. On average, only 45.74% of the total reads of a sample were successfully classified at the species level by MSR with reference to the Greengenes database. After merging paired-end reads and quality filtering, 94.02% of filtered reads were classified at the species level by Illumina_BLAST+ with reference to the NCBI 16S rRNA database.

In the Nanopore workflow, both Epi2me and NanoBLAST+ use the NCBI 16S rRNA database for classification of long-read sequencing data. An average of 76.03% of total reads were classified at the species level in Epi2me, compared with 53.56% in NanoBLAST+.

### Concordance in bacterial speciation: Illumina 16S and Nanopore 16S versus Sanger 16S.

**FIG 2:**
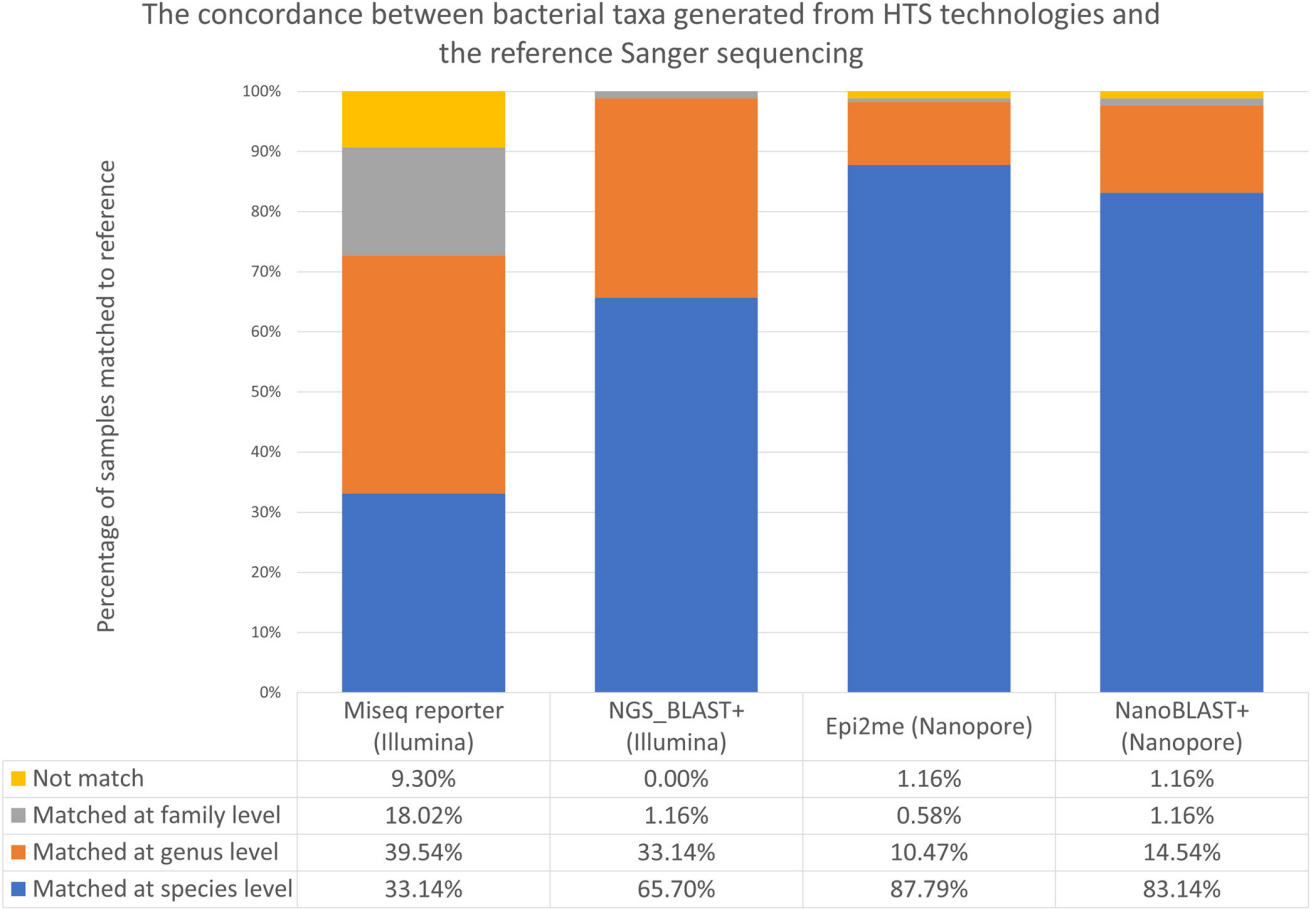
Concordance between bacterial taxa inferred by the two HTS workflows and Sanger sequencing.

**FIG 3:**
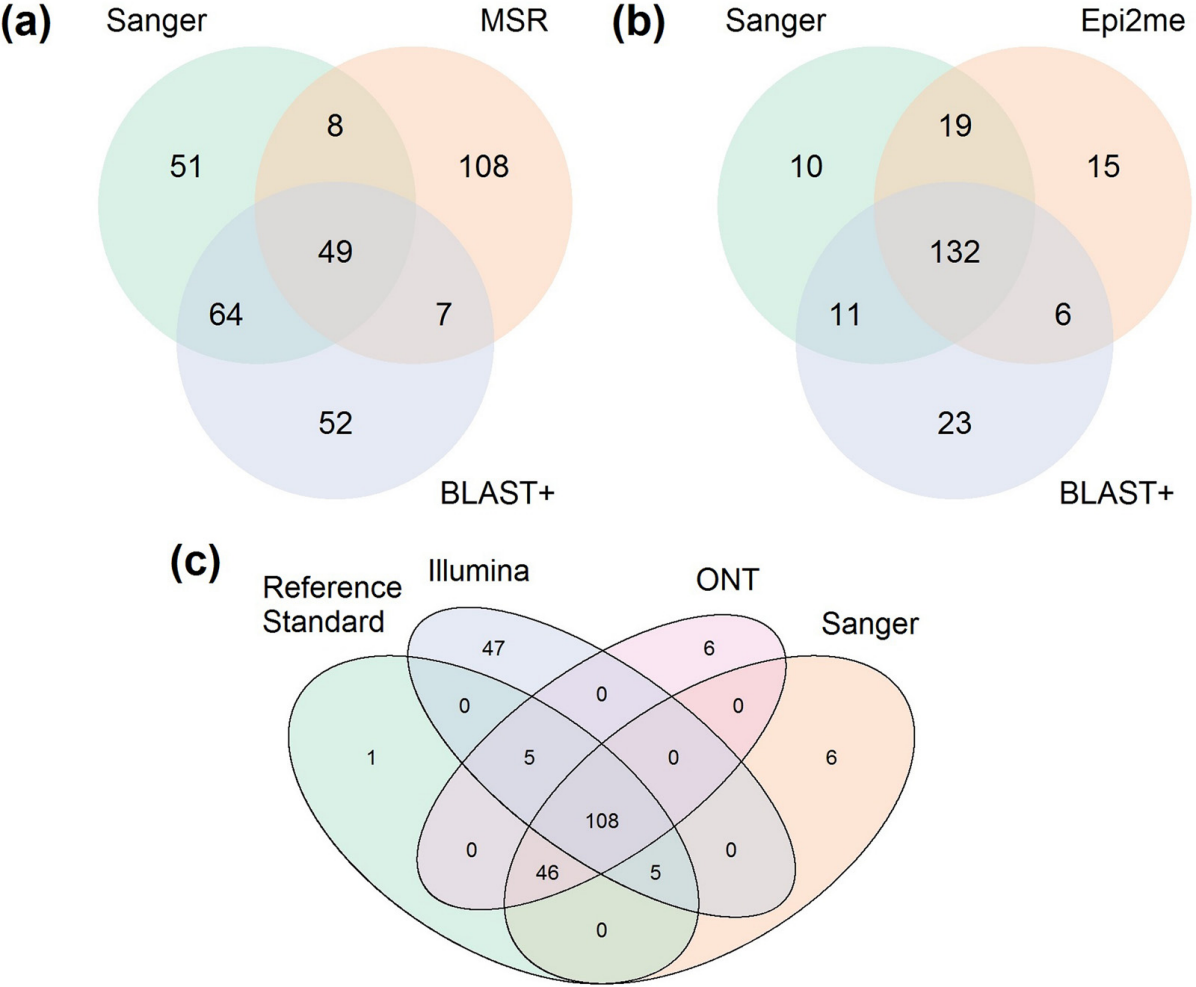
Venn diagram showing concordance of bacterial taxa inferred by different 16S rRNA sequencing platforms. (a) Concordance of the top classified species between Illumina sequencing, coupled with MSR and Illumina_BLAST+ analysis, and Sanger sequencing. (b) Concordance of the top classified species between Nanopore sequencing, coupled with Epi2ME and nanoBLAST+, and Sanger sequencing. (c) Concordance of the top classified species among Sanger 16S, resolved Illumina 16S, resolved Nanopore 16S, and reference standard.

The top-ranked species obtained from the Illumina 16S and Nanopore 16S workflows, coupled with the respective analysis pipelines, are listed in Table S3. The percentage of samples that matched Sanger 16S results at each of the species, genus, and family levels is illustrated in Fig. 2. The concordance in species-level identification among the sequencing platforms is shown in Fig. 3. Overall, in terms of concordance with the Sanger 16S result, Nanopore 16S was better than Illumina 16S, regardless of analysis pipeline.

For the Illumina 16S workflow, MSR and Illumina_BLAST+ demonstrated concordances of 33.14% (57/172) and 65.70% (113/172), respectively, with Sanger 16S in species-level identification. A total of 9.30% of samples (16/172) were unmatched, even at the family level, in MSR, whereas all samples matched at the family level or below in Illumina_BLAST+. Of note, concordance between the results of MSR and Illumina_BLAST+ was low; only 32.56% of samples (56/172) showed a matched result among the classified species from these two analysis pipelines. Moreover, only 28.49% of samples (49/172) showed complete agreement in the classified species among the MSR, Illumina_BLAST+, and Sanger data sets.

For the 116 samples with discrepant taxa inferred by MSR and Illumina_BLAST+, nf-core/ampliseq was used to resolved their identities. However, only 41 samples were classified at the species level by nf-core/ampliseq, 28 (24.14%) of them matched the results of Illumina_BLAST+, and 4 (3.45%) of them matched the results of MSR. For the nine samples that failed to reach agreement at the species level, all of them matched the results of Illumina_BLAST+ at the genus level. A total of 75 samples were classified only at the genus level or above by nf-core/ampliseq, and all of them matched the genus or family inferred by Illumina_BLAST+. Concordance between the resolved Illumina 16S and Sanger 16S results was 63.95% (110/172).

For Nanopore 16S, concordances of 87.79% (151/172) and 83.14% (143/172) at the species level were achieved with Epi2me and NanoBLAST+, respectively. A total of 1.16% of samples (2/172) were unmatched even at the family level, as reported by Epi2me and NanoBLAST+, respectively. Concordance between the results of Epi2me and NanoBLAST+ was 80.23% (138/172). Additionally, 76.74% of samples (132/172) showed agreement in the classified species among the Epi2me, NanoBLAST+, and Sanger data sets.

A total of 34 samples showed disagreement in the classified species inferred by Epi2me and NanoBLAST+. The respective Nanopore data were further analyzed using NanoCLUST to resolve the discrepancies. NanoCLUST agreed with Epi2ME and BLAST+ in 13 (38.24%) and 17 (50.00%) samples, respectively. Four samples failed to reach agreement in terms of species-level identification, of which three were matched in terms of genus-level identification and one was considered as having no reliable bacterial identification. Concordance between the resolved Nanopore 16S and Sanger 16S results was 89.53% (154/172).

### WGS for bacterial isolates with discrepant species-level identification.

Eight samples (4.65% [8/172]) showed complete discordance in bacterial species, as inferred by the three 16S rRNA sequencing workflows. WGS was conducted to identify definite taxa. To validate the transposase-based rapid sequencing protocol for bacterial genome construction, two ATCC reference strains, namely, Klebsiella pneumoniae BAA3079 and Staphylococcus aureus BAA3114, were sequenced and analyzed in parallel with the eight discordant samples. Both reference strains successfully yielded consensus sequences of >3 Mb, which covered 94% of the genomes of the respective target organisms with 99% identity. This indicated that the WGS protocol was able to construct reliable consensus prokaryotic genomes (Table 1).

**TABLE 1:** WGS analysis for samples with completely discordant taxonomic assignment by Sanger, Illumina, and Nanopore 16S rRNA sequencing^*a*^

Sample ID	Species inferred by Sanger 16S	Species inferred by resolved Illumina 16S^*c*^	Species inferred by resolved Nanopore 16S^*d*^	WGS results						
				Best-matched species by WGS (reference genome accession no.)	Shasta genome assembly			Miniasm genome assembly		
					Query coverage (%)	Identity (%)	ANI (%)^*e*^	Query coverage (%)	Identity (%)	ANI (%)^*e*^
Klebsiella pneumoniae BAA3079^*b*^	NA	NA	NA	Klebsiella pneumoniae (NC_016845.1)	99.00	97.00	98.92	92.13	99.40	99.14
Staphylococcus aureus BAA3114^*b*^	NA	NA	NA	Staphylococcus aureus (NC_007795.1)	94.06	99.95	99.30	88.39	99.92	99.23
R001	Kocuria koreensis	*Kocuria massiliensis*	*Kocuria* spp.	*Kocuria massiliensis* (NZ_LT835161.1)	42.21	87.44	78.29	42.42	87.41	78.55
R006	*Kocuria koreensis*	*Kocuria massiliensis*	*Kocuria* spp.	*Kocuria massiliensis* (NZ_LT835161.1)	43.04	79.12	78.49	42.04	87.49	78.44
R062	Klebsiella grimontii	Enterobacter cloacae	Yokenella regensburgei	Klebsiella michiganensis (NZ_CP060111.1)	92.17	99.17	98.71	86.30	98.99	98.69
R120	Brachybacterium conglomeratum	Brachybacterium faecium	Brachybacterium paraconglomeratum	Brachybacterium saurashtrense (NZ_CP031356.1)	62.15	85.18	82.30	62.30	85.12	82.39
R121	Schaalia odontolytica	Schaalia vaccimaxillae	Sphingomonas paucimobilis	*Schaalia odontolytica* (NZ_CP046315.1)	6.07	78.55	70.34	6.04	78.24	70.86
R131	*Schaalia odontolytica*	*Schaalia vaccimaxillae*	No reliable ID	*Schaalia odontolytica* (NZ_CP046315.1)	6.19	82.12	71.21	6.29	78.25	71.26
R158	Microbacterium ginsengiterrae	Microbacterium assamensis	Microbacterium foliorum	Microbacterium foliorum (NZ_CP041040.1)	65.41	84.52	82.24	65.21	84.51	82.15
R181	Sphingomonas yabuuchiae	Sphingomonas paucimobilis	Sphingomonas sanguinis	Sphingomonas hominis (NZ_JABULH010000007.1)	31.48	89.67	82.09	30.68	89.59	81.95

a ID, identification; NA, not applicable.

b Klebsiella pneumoniae BAA3079 and Staphylococcus aureus BAA3114 served as quality control samples, which were sequenced and analyzed in parallel with the discordant samples for WGS and bioinformatics analysis.

c Discordant samples between MSR and Illumina_BLAST+ were resolved by nf-core/ampliseq.

d Discordant samples between Epi2me and NanoBLAST+ were resolved by NanoCLUST.

e An average nucleotide identity (ANI) of >94% indicated that the samples belonged to the same species as the best-matched genomes.

Interestingly, seven of these samples failed to match the published bacterial genomes, with query coverage of <70% for the longest consensus sequences (Table 1). The average nucleotide identities (ANIs) to the best-matched genomes were <85% (the threshold for the same species should be >94%), suggesting that these seven “difficult-to-identify” isolates were likely novel bacterial species. As the definite bacterial species could not be confirmed, these samples were excluded from the subsequent diagnostic evaluation.

The consensus sequence of one sample (R062) showed an overall query coverage of >92%, with 99.17% identity to Klebsiella michiganensis (NZ_CP060111.1). As the ANI achieved 98.71%, *K. michiganensis* was therefore considered the reference taxon for this sample.

### Diagnostic accuracy of the three 16S rRNA sequencing workflows.

The composite of 16S rRNA sequencing and WGS results was regarded as the reference standard for calculating the diagnostic accuracy. The discordant samples between each sequencing platform and the reference standards are listed in Table 2.

**TABLE 2:** Samples with mismatched taxa inferred by at least one sequencing platform

Sample ID	Species-level ID (reference standard)	Sanger sequencing		Illumina sequencing		Nanopore sequencing	
		Classified species from Sanger 16S^*a*^	16S identity against the reference (%)	Classified species from resolved Illumina 16S^*a*^	16S identity against the reference (%)	Classified species from resolved Nanopore 16S^*a*^	16S identity against the reference (%)
R003	Pseudoglutamicibacter albus	Pseudoglutamicibacter cumminsii	99.26	Pseudoglutamicibacter albus	Matched	Pseudoglutamicibacter albus	Matched
R013	Microbacterium hominis	Microbacterium hominis	Matched	Microbacterium aerolatum	97.47	Microbacterium hominis	Matched
R017	Microbacterium hominis	Microbacterium hominis	Matched	Microbacterium aerolatum	97.47	Microbacterium hominis	Matched
R021	Microbacterium hominis	Microbacterium hominis	Matched	Microbacterium aerolatum	97.47	Microbacterium hominis	Matched
R024	Bacillus idriensis	Bacillus idriensis	Matched	Bacillus idriensis	Matched	Bacillus indicus	97.62
R025	Varibaculum cambriense	Varibaculum cambriense	Matched	Varibaculum anthropi	98.50	Varibaculum cambriense	Matched
R026	Varibaculum cambriense	Varibaculum cambriense	Matched	Varibaculum anthropi	98.50	Varibaculum cambriense	Matched
R036	Corynebacterium lowii	Corynebacterium lowii	Matched	Corynebacterium bovis	93.29	Corynebacterium lowii	Matched
R040	Weissella cibaria	Weissella cibaria	Matched	Weissella confusa	99.26	Weissella cibaria	Matched
R043	Proteus vulgaris	Proteus vulgaris	Matched	Proteus alimentorum	99.64	Proteus vulgaris	Matched
R045	Brucella microti	Brucella microti	Matched	Brucella papionis	99.86	Brucella microti	Matched
R047	Proteus cibarius	Proteus cibarius	Matched	Proteus terrae	99.65	Proteus cibarius	Matched
R049	Dermacoccus barathri	Dermacoccus barathri	Matched	Dermacoccus profundi	99.86	Dermacoccus barathri	Matched
R052	Arcanobacterium wilhelmae	Arcanobacterium wilhelmae	Matched	Arcanobacterium pinnipediorum	96.60	Arcanobacterium wilhelmae	Matched
R053	Dermacoccus barathri	Dermacoccus barathri	Matched	Dermacoccus profundi	99.86	Dermacoccus barathri	Matched
R056	Corynebacterium simulans	Corynebacterium simulans	Matched	Corynebacterium glutamicum	93.74	Corynebacterium simulans	Matched
R058	Corynebacterium mastitidis	Corynebacterium mastitidis	Matched	Corynebacterium tuberculostearicum	94.67	Corynebacterium mastitidis	Matched
R062	Klebsiella michiganensis	Klebsiella * grimontii*	99.20	Enterobacter cloacae	97.07	Yokenella regensburgei	98.56
R063	Corynebacterium pilbarense	Corynebacterium pilbarense	Matched	Corynebacterium coyleae	98.04	Corynebacterium pilbarense	Matched
R069	Eikenella corrodens	Eikenella corrodens	Matched	Eikenella halliae	98.69	Eikenella corrodens	Matched
R071	Corynebacterium xerosis	*Corynebacterium hansenii*	99.07	Corynebacterium xerosis	Matched	Corynebacterium xerosis	Matched
R072	Mycolicibacterium fortuitum	*Mycolicibacterium fortuitum*	Matched	Mycolicibacterium arcueilense	98.96	*Mycolicibacterium fortuitum*	Matched
R073	Tessaracoccus oleiagri	Tessaracoccus oleiagri	Matched	Tessaracoccus flavescens	95.95	Tessaracoccus oleiagri	Matched
R078	Vagococcus teuberi	*Vagococcus teuberi*	Matched	Vagococcus martis	99.22	*Vagococcus teuberi*	Matched
R079	Corynebacterium xerosis	*Corynebacterium hansenii*	99.07	Corynebacterium xerosis	Matched	Corynebacterium xerosis	Matched
R083	Tessaracoccus oleiagri	Tessaracoccus oleiagri	Matched	Tessaracoccus flavescens	95.95	Tessaracoccus oleiagri	Matched
R086	Raoultella planticola	Raoultella planticola	Matched	Raoultella planticola	Matched	Klebsiella aerogenes	99.06
R094	Corynebacterium xerosis	Corynebacterium hansenii	99.07	Corynebacterium xerosis	Matched	Corynebacterium xerosis	Matched
R096	Streptomyces thermodiastaticus	Streptomyces thermodiastaticus	Matched	Streptomyces thermoviolaceus	98.86	Streptomyces thermodiastaticus	Matched
R097	Pseudoxanthomonas helianthi	Pseudoxanthomonas helianthi	Matched	Pseudoxanthomonas spadix	97.04	Pseudoxanthomonas helianthi	Matched
R098	Brachybacterium huguangmaarense	Brachybacterium huguangmaarense	Matched	Brachybacterium huguangmaarense	Matched	Brachybacterium nesterenkovii	97.84
R104	Gordonia sputi	Gordonia sputi	Matched	Gordonia otitidis	99.07	Gordonia sputi	Matched
R105	Gordonia sputi	Gordonia sputi	Matched	Gordonia otitidis	99.07	Gordonia sputi	Matched
R107	Moraxella osloensis	Moraxella osloensis	Matched	Enhydrobacter aerosaccus	99.19	Moraxella osloensis	Matched
R108	Staphylococcus saccharolyticus	Staphylococcus saccharolyticus	Matched	Staphylococcus epidermidis	99.19	Staphylococcus saccharolyticus	Matched
R112	Citrobacter sedlakii	Citrobacter sedlakii	Matched	Citrobacter youngae	98.32	Citrobacter sedlakii	Matched
R116	Tsukamurella tyrosinosolvens	Tsukamurella tyrosinosolvens	Matched	*Tsukamurella ocularis*	99.86	Tsukamurella tyrosinosolvens	Matched
R123	Pseudoglutamicibacter albus	Pseudoglutamicibacter cumminsii	99.26	Pseudoglutamicibacter albus	Matched	Pseudoglutamicibacter albus	Matched
R133	Nocardia brasiliensis	Nocardia brasiliensis	Matched	Nocardia vulneris	99.31	Nocardia brasiliensis	Matched
R140	Moraxella lacunata	Moraxella lacunata	Matched	Moraxella equi	99.38	Moraxella lacunata	Matched
R141	Ottowia beijingensis	Ottowia beijingensis	Matched	Brachymonas denitrificans	93.33	Ottowia beijingensis	Matched
R148	Moraxella osloensis	Moraxella osloensis	Matched	Enhydrobacter aerosaccus	99.19	Moraxella osloensis	Matched
R149	Ornithinibacillus californiensis	Ornithinibacillus californiensis	Matched	Ornithinibacillus scapharcae	98.48	Ornithinibacillus californiensis	Matched
R151	Dermacoccus barathri	Dermacoccus barathri	Matched	Dermacoccus profundi	99.86	Dermacoccus barathri	Matched
R153	Corynebacterium mastitidis	Corynebacterium mastitidis	Matched	Corynebacterium tuberculostearicum	94.67	Corynebacterium mastitidis	Matched
R167	Moraxella osloensis	Moraxella osloensis	Matched	Enhydrobacter aerosaccus	99.19	Moraxella osloensis	Matched
R175	Corynebacterium pollutisoli	Corynebacterium pollutisoli	Matched	Corynebacterium humireducens	98.07	Corynebacterium pollutisoli	Matched
R176	Tsukamurella oculari *s*	*Tsukamurella ocularis*	Matched	*Tsukamurella ocularis*	Matched	Tsukamurella hominis	100.00
R178	Acinetobacter soli	Acinetobacter soli	Matched	Acinetobacter soli	Matched	Acinetobacter lactucae	97.82
R179	Corynebacterium lipophiloflavum	Corynebacterium lipophiloflavum	Matched	Corynebacterium mycetoides	97.16	Corynebacterium lipophiloflavum	Matched
R180	Corynebacterium mastitidis	Corynebacterium mastitidis	Matched	Corynebacterium tuberculostearicum	94.67	Corynebacterium mastitidis	Matched
R182	Fusobacterium nucleatum	Fusobacterium nucleatum	Matched	Fusobacterium canifelinum	98.34	Fusobacterium nucleatum	Matched
R183	Parabacteroides faecis	Parabacteroides faecis	Matched	Parabacteroides chongii	97.15	Parabacteroides faecis	Matched
R190	Bacillus xiamenensis	Bacillus xiamenensis	Matched	Bacillus aerius	97.16	Bacillus xiamenensis	Matched
R192	Corynebacterium pilbarense	Corynebacterium pilbarense	Matched	Corynebacterium ureicelerivorans	98.85	Corynebacterium pilbarense	Matched
R204	Prevotella scopos	Prevotella scopos	Matched	Prevotella melaninogenica	98.10	Prevotella scopos	Matched
R205	Pasteurella multocida	Pasteurella multocida	Matched	Pasteurella stomatis	93.74	Pasteurella multocida	Matched
R206	Staphylococcus cohnii	Staphylococcus cohnii	Matched	Staphylococcus auricularis	98.16	Staphylococcus cohnii	Matched
R208	Achromobacter denitrificans	Achromobacter denitrificans	Matched	Achromobacter xylosoxidans	99.15	Achromobacter denitrificans	Matched
R210	Bacillus licheniformis	Bacillus licheniformis	Matched	*Bacillus piscis*	97.37	Bacillus licheniformis	Matched

a Mismatched taxa are underlined. Sanger, Illumina, and Nanopore 16S rRNA sequencing results are shown.

The diagnostic performance of each sequencing workflow is summarized in Table 3. For the Illumina platform, the diagnostic accuracies of MSR and Illumina_BLAST+ were 35.76% and 71.52%, respectively. Notably, the diagnostic accuracy of resolved Illumina 16S was even lower than that of Illumina_BLAST+ alone (69.07% versus 71.52%), suggesting that Illumina_BLAST+ was the most optimized analysis pipeline for Illumina 16S.

**TABLE 3:** Diagnostic accuracies of the Sanger, Illumina, and Nanopore 16S rRNA sequencing methods

Sequencing method	No. of samples analyzed	No. of samples with matched taxa	Diagnostic accuracy (%)	95% CI^*c*^	*P* value (chi-square test)^*d*^
Sanger 16S	165	159	96.36	92.25–98.65	
Resolved Illumina 16S^*a*^	165	115	69.70	62.07–76.60	<0.0001*
Analyzed by MSR	165	59	35.76	28.46–43.58	
Analyzed by Illumina_BLAST+	165	118	71.52	63.98–78.26	
Resolved Nanopore 16S^*b*^	165	159	96.36	92.25–98.65	0.0291*
Analyzed by Epi2ME	165	147	89.09	83.31–93.41	
Analyzed by NanoBLAST+	165	148	89.70	84.02–93.88	

a Discordant samples between MSR and Illumina_BLAST+ were analyzed by nf-core/ampliseq; classified species in nf-core/ampliseq were considered resolved identities in Illumina workflow.

b Discordant samples between Epi2me and NanoBLAST+ were analyzed by NanoCLUST; classified species in NanoCLUST were considered resolved identities in Nanopore workflow.

c CI, confidence interval.

d *, *P* < 0.05, statistically significantly different from Sanger 16S results.

For the Nanopore platform, the diagnostic accuracies of Epi2me and nanoBLAST+ were 89.09% and 89.70%, respectively. The diagnostic accuracy of resolved Nanopore 16S was 96.36%, which was the same as that of Sanger sequencing.

### Comparison of sample-to-report time and running cost of the two HTS technologies.

**FIG 4:**
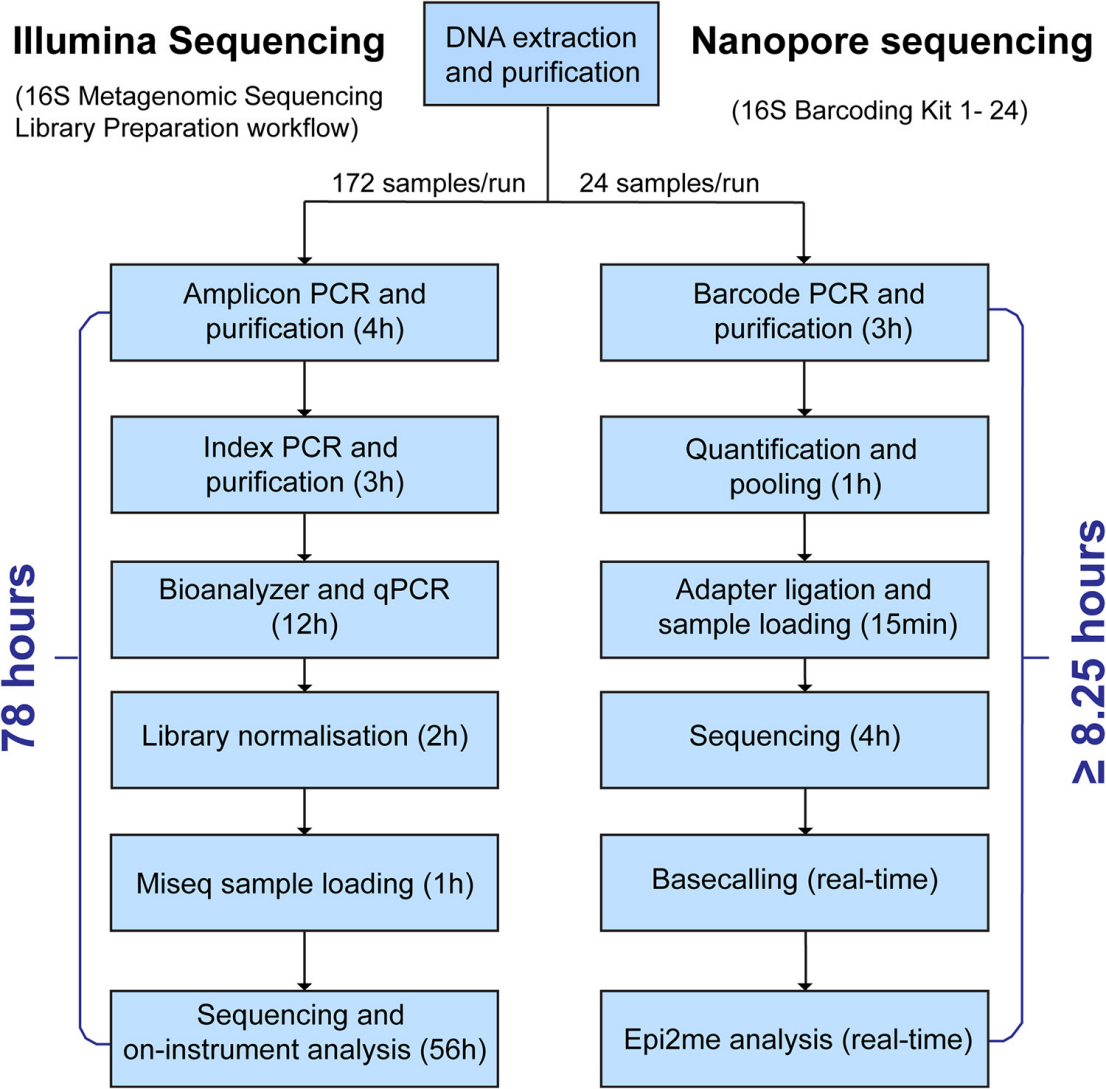
16S rRNA gene sequencing workflow of the HTS technologies.

The Illumina platform enables sequencing of up to 384 samples per run, whereas, owing to the limited choice of sequencing barcodes, the Nanopore platform can support only a batch of 24 samples per run. Without considering the time for DNA extraction, it took 78 h for the Illumina workflow to generate sequencing data for each run (Fig. 4). With the Nanopore platform, the sequencing workflow required 8.25 h. Of note, although base-calling and Epi2me analyses are real-time processes, their speed is highly dependent on the strength of the computer. However, Nanopore sequencing can be stopped once sufficient reads have been generated.

The running cost of the Nanopore workflow is relatively lower than that of the Illumina workflow. The cost of the Illumina workflow per sequencing run is US$4,931 (172 samples), and the cost per sample is approximately US$28.7. If the sample size is increased to 384, the cost of the Illumina workflow per sequencing run is US$8,279; therefore, the cost per sample is reduced to US$21.6. For the Nanopore workflow, the cost per sequencing run (24 samples) is US$424, which means that the cost per sample is approximately US$17.7.

## DISCUSSION

Although the majority of bacterial pathogens can be identified by MALDI-TOF MS, 16S rRNA gene sequencing is needed in clinical microbiology laboratories to confirm the identities of “difficult-to-identify” clinical isolates. With reduced costs, simplified protocols, and automated bioinformatics pipelines, HTS has been proposed as a better alternative to traditional Sanger sequencing for sequence-based bacterial identification in clinical laboratories. This is the first study to compare the performances and evaluate the clinical utilities of two commercially available high-throughput 16S rRNA gene sequencing assays with built-in analysis software for taxonomic assignment of bacterial pathogens that are unidentifiable using MALDI-TOF MS.

**FIG 5:**
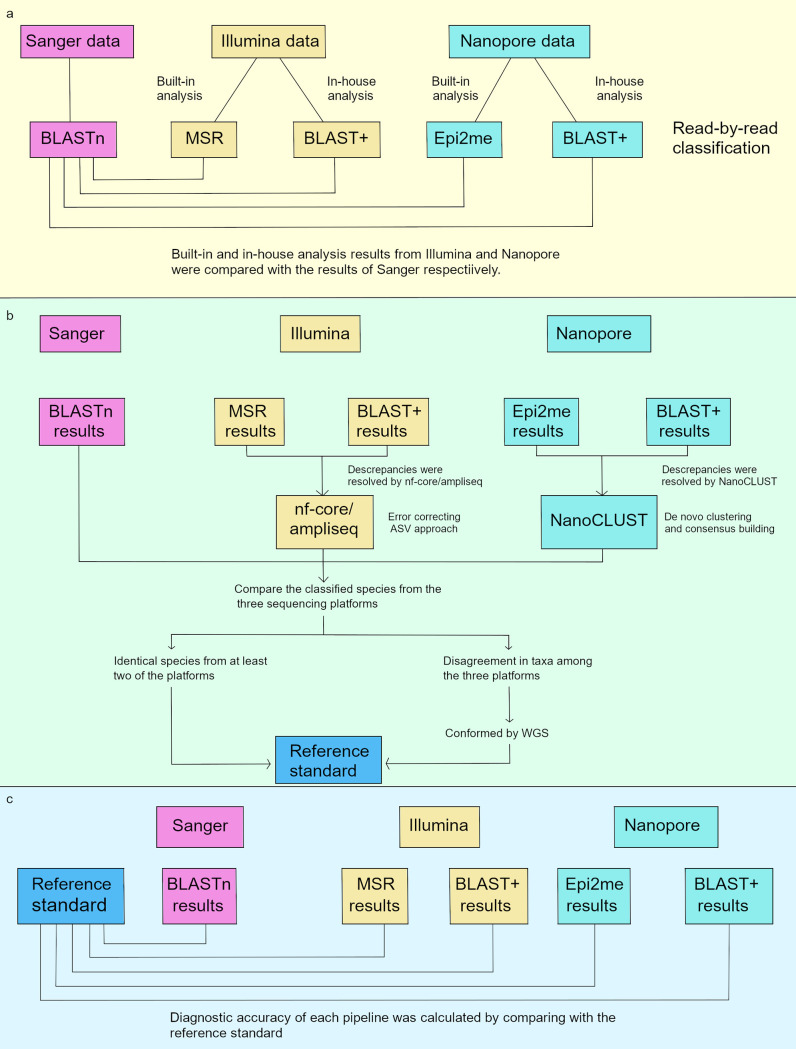
Analysis workflow of sequencing data from each platform. (a) Results from Illumina and Nanopore platforms are compared to the Sanger 16S result. (b) Composite reference standard. (c) Calculating diagnostic accuracy.

In order to evaluate the performance of the built-in analysis pipelines from Illumina (MSR) and Nanopore (Epi2me) platforms, the sequencing data from both platforms were also analyzed using BLAST+. With the same analysis approach as that of MSR and Epi2me (read-by-read classification) and the applicability to both Illumina and Nanopore data, BLAST+ is a good analysis tool for intra- and interplatform comparisons. The full analysis workflow is illustrated in Fig. 5.

The results from Illumina and Nanopore platforms were compared with Sanger 16S results (Fig. 5a). With the Illumina platform, the concordance of the classified species between MSR and Sanger 16S was exceptionally low; only 33.14% of samples matched the Sanger result for the top classified species, compared with 65.70% when using Illumina_BLAST+. As described in previous studies, the use of different bioinformatics tools and 16S rRNA sequence databases could result in different taxonomic assignments, especially at lower taxonomic levels (26, 27). The latest version of the Greengenes database for MSR was updated in 2013 and does not contain certain new bacterial taxa, which accounts for the poor agreement of this workflow compared with others (27). Nevertheless, mismatches between Illumina and Sanger sequencing were observed in 34.33% of samples, even when the same aligner (i.e., BLAST) and database (i.e., NCBI 16S bacterial database) were used.

The Nanopore 16S workflow demonstrated a considerably higher percentage concordance with the Sanger 16S workflow than with the Illumina 16S workflow, regardless of the analysis pipeline used. In contrast to the built-in analysis on the Illumina platform (i.e., MSR), the performance of Epi2me with Nanopore 16S was comparable to that of nanoBLAST+ (83.14%), with 87.79% of samples matching the Sanger results for the top classified species. Notably, species-level disagreement between Epi2me and nanoBLAST+ was observed in 34 samples (19.77%).

One may argue that with the constraint of low sequencing depth, the Sanger 16S result alone should not be considered as the final reference. We therefore used a composite of 16S sequencing results generated by the three platforms, and any discrepancies were resolved by WGS as the reference standard to determine the diagnostic accuracy of the HTS workflows (Fig. 5b and c).

The discrepant samples between MSR and Illumina_BLAST+ were further analyzed by nf-core/ampliseq. This new pipeline classifies reads based on an error-correcting amplicon sequence variant (ASV) approach, which showed better performance in taxonomic classification than the clustering of operational taxonomic unit (OTU) approach in the study by Straub et al. (21). However, there was no improvement in the diagnostic accuracy when the resolved Illumina 16S was compared with the reference standards. Regardless of the classification approaches, the diagnostic accuracy of the Illumina workflow was still restricted by the length and position of the variable regions of the 16S gene fragment being sequenced.

As indicated by Johnson et al., although some subregions (e.g., V1 to V3) of the 16S rRNA gene provide a reasonable approximation of 16S diversity, most do not capture sufficient sequence variation to discriminate between closely related taxa. Also, different subregions show bias in the bacterial taxa that can be identified (28). In this study, V3 and V4 regions might perform poorly in classifying the genera of discordant samples (Table 2) down to the species level. However, Illumina_BLAST+ showed a high concordance to the reference at the genus level (98.79%), meaning that the genus-level identification of the Illumina platform is credible.

Epi2me and BLAST+ rely on read-by-read alignment to reference sequences in the database. As the base-calling accuracy of Nanopore sequencing is relatively low, the prevalence of sequencing errors in Nanopore reads could limit its ability to resolve highly similar sequences. Alternatively, NanoCLUST generates clusters based on uniform manifold approximation and projection (UMAP) and classifies the representative consensus read in each cluster using BLAST. The effect of sequencing errors in individual sequences can be minimized by forming clusters, which reduces the chance of misclassification. Comparing the species resolved using NanoCLUST with the reference standard, there was a slight improvement in diagnostic accuracy from 89.09% (Epi2me) and 89.70% (nanoBLAST+) to 96.36%.

There were six samples (3.64%) that still failed to match the reference at the species level for the resolved Nanopore 16S. One possible reason for this discordance is the high similarity in 16S rRNA gene sequences between the inferred species and the reference taxa. Based on the now historic assumption of 16S rRNA sequencing, sequences with >95% identity represent the same genus, whereas sequences with >97% identity represent closely related species (29). Many researchers have reported that the taxonomic resolution of the 16S rRNA gene is lower and is unable to discriminate the closely related species in certain genera, including but not limited to *Bacillus*, *Burkholderia*, Acinetobacter baumannii*-calcoaceticus* complex, *Achromobacter*, *Actinomyces*, and Staphylococcus and *Enterobacterales* (30, 31). In this study, all six taxa inferred by Nanopore 16S had >97% sequence identity with the reference standard (Table 2).

In addition, WGS was performed to identify the definite bacterial taxa for the eight samples with completely discordant 16S results given by three sequencing platforms. Nonetheless, seven samples were considered novel bacterial species due to the low query coverage (<50%) and low ANIs (<94%) between the respective consensus sequence and best-matched genome (32). WGS confirmed that R062 belonged to *K. michiganensis* (ANI = 98.71%), which shared a high degree of 16S rRNA identity with the taxa assigned by Sanger 16S (Klebsiella grimontii; 99.20%), resolved Illumina 16S (Enterobacter cloacae; 97.07%), and resolved Nanopore 16S (Yokenella regensburgei; 98.56%) (Table 1). This demonstrated that 16S rRNA sequencing was not able to accurately differentiate these closely related species.

Considering the time to result (not including DNA extraction) of the two sequencing platforms, the Nanopore workflow (8.25 h) has a much shorter turnaround time than the Illumina workflow (78 h). A long quantification process (quantitative PCR [qPCR] and bioanalyzer) is required in the Illumina workflow (12 h) since the cluster generation process in Illumina sequencing is highly sensitive to library concentration. While overclustering leads to lower base accuracy, underclustering leads to lower data output in Illumina sequencing. In contrast, Nanopore sequencing is less sensitive to the fluctuation of library concentration, and the DNA quantification process is simpler.

The largest sample size of the Nanopore 16S workflow is 24 samples per batch, compared to 384 samples per batch in the Illumina 16S workflow. Comparing the cost per sample in a sequencing run with respective maximum sample size, Nanopore sequencing is relatively cheaper than Illumina sequencing (US$17.7 versus US$21.6, respectively). Additionally, the startup cost of Nanopore sequencing is remarkably lower than that of Illumina sequencing. The starter package of Nanopore sequencing costs only US$1,000, whereas the Illumina MiSeq costs approximately US$125,000. Also, expensive instruments like a qPCR machine and a bioanalyzer are required for the quantification step in Illumina sequencing.

In this study, the FLO-MIN106 R9.4.1 reusable flow cell, which enables sequencing for up to 72 h, was used for Nanopore 16S sequencing. However, library carryover from the previous run was observed in a pilot study. This is problematic when the same barcode set is used in consecutive sequencing runs. To avoid contamination by library carryover, a new flow cell was used in each sequencing run, and used flow cells were reserved for other sequencing runs using different barcodes. In this context, the disposable Flongle flow cell with fewer active pores is preferred in a clinical setting, especially when the sample size is small.

Bacterial identification at the genus level might be enough for prescribing treatment in some cases, since most antimicrobial drugs act against groups of bacteria instead of single species. However, identification to the species level is crucial in differentiating environmental nonpathogenic species and pathogenic species, especially when the bacteria have contrasting drug susceptibility patterns, for example, the A. calcoaceticus-A. baumannii complex (33). Nevertheless, the taxonomic resolution of 16S sequencing is dependent on the read length of the 16S rRNA gene, the capacity of the 16S reference database, and the choice of analysis pipeline.

There are some limitations to this study. First, the aim of this study was to compare commercially available kits for 16S rRNA gene sequencing from Illumina and Nanopore. Therefore, by using the 16S metagenomic sequencing library preparation kit, only the V3 and V4 subregions of the 16S rRNA gene were sequenced in the Illumina workflow. But, it is possible to sequence the full-length 16S rRNA gene using Illumina MiSeq with a laboratory-developed protocol (31), which may increase the diagnostic accuracy of the Illumina workflow. However, the analysis is more complicated since an additional step of making contigs is required, which could not be done by MSR. Second, except for the eight discordant samples, the reference taxa of isolates were defined solely by 16S rRNA sequencing, and it may not represent the definite taxa. Third, the taxonomic assignment in WGS was based on the contigs of consensus sequences after *de novo* assembly. Circular, gap-free bacterial genomes were not constructed.

### Conclusions.

Because of its rapidity, simplicity, and high accuracy, MALDI-TOF MS is the mainstay of bacterial identification in clinical microbiology laboratories. 16S sequencing of cultured isolates should only be used for taxonomic assignment of unidentifiable bacterial pathogens in MALDI-TOF MS.

The performance of MSR in taxonomic classification was unsatisfactory, and analysis using external pipelines such as BLAST+ was recommended in the Illumina 16S workflow (Nextera XT index kit v2). With massive throughput and high base accuracy, the Illumina platform is suitable for clinical laboratories with a high burden of clinical samples, where a longer turnaround time is acceptable. The Nanopore 16S workflow (SQK-16S024 with Epi2me) is recommended when rapid species-level identification is required, especially in emergency cases. It is recommended to further confirm the classified species using other analysis pipelines in both sequencing platforms to increase the diagnostic accuracy.
