# Supplementary material for: The Clinical Utility of Two High-Throughput 16S rRNA Gene Sequencing Workflows for Taxonomic Assignment of Unidentifiable Bacterial Pathogens in Matrix-Assisted Laser Desorption Ionization–Time of Flight Mass Spectrometry
Source: J Clin Microbiol. 2022 Jan 19;60(1):e01769-21. doi: 10.1128/JCM.01769-21 (PMC8769742; doi:10.1128/JCM.01769-21)
Supplement: Supplemental file 2 — Fig. S1. Download JCM.01769-21-s0002.pdf, PDF file, 0.9 MB [file jcm.01769-21-s0002.pdf]

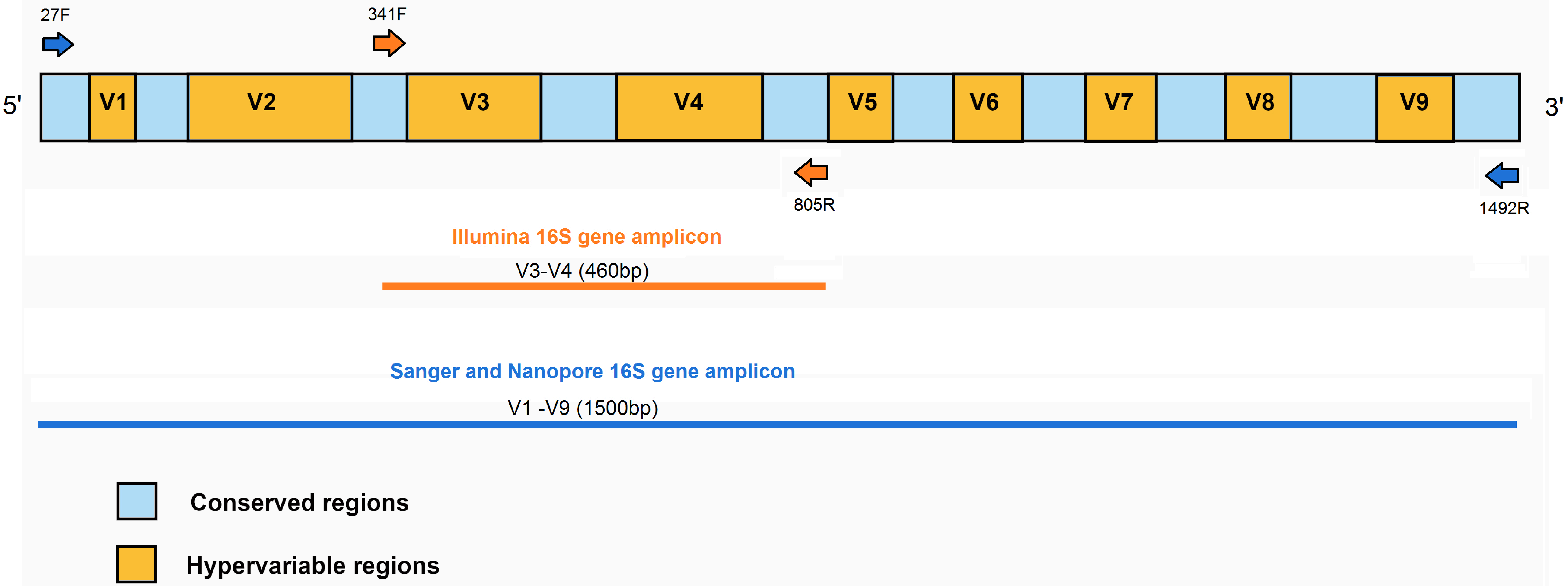

Supplementary figure 1. The 16S gene amplicon regions of Sanger, Illumina and Nanopore sequencing workflows.
